# Examining the effect of universal testing and treatment strategies for HIV prevention in Zambia and South Africa: generalizing the results of the HPTN 071 (PopART) trial

**DOI:** 10.1002/jia2.70062

**Published:** 2025-11-26

**Authors:** Bonnie E. Shook‐Sa, Paul N. Zivich, Stephen R. Cole, Nora E. Rosenberg, Michael G. Hudgens, Deborah J. Donnell, Sizulu Moyo, Khangelani Zuma, Helen Ayles, Peter Bock, Joseph J. Eron, Richard J. Hayes, Jessie K. Edwards

**Affiliations:** ^1^ Department of Biostatistics University of North Carolina at Chapel Hill Chapel Hill North Carolina USA; ^2^ Nuffield Department of Population Health University of Oxford Oxford UK; ^3^ Department of Epidemiology University of North Carolina at Chapel Hill Chapel Hill North Carolina USA; ^4^ Department of Health Behavior University of North Carolina at Chapel Hill Chapel Hill North Carolina USA; ^5^ Fred Hutchinson Cancer Research Center Seattle Washington USA; ^6^ Human Sciences Research Council Pretoria South Africa; ^7^ School of Public Health and Family Medicine University of Cape Town Cape Town South Africa; ^8^ London School of Hygiene and Tropical Medicine London UK; ^9^ Desmond Tutu TB Centre Department of Paediatrics and Child Health Stellenbosch University Stellenbosch South Africa; ^10^ Division of Infectious Diseases University of North Carolina at Chapel Hill Chapel Hill North Carolina USA

**Keywords:** HIV, universal testing and treatment, prevention, external validity, PopART, generalizability

## Abstract

**Introduction:**

HIV Prevention Trials Network (HPTN) 071 (PopART) was a cluster‐randomized trial to evaluate universal testing and treatment (UTT) strategies for HIV prevention. HPTN071 compared three arms: (A) combination prevention with UTT; (B) combination prevention with universal testing and antiretroviral therapy initiation according to local guidelines; and (C) standard of care (SOC). Interventions were implemented in entire randomized communities, with impacts on HIV incidence measured in “population cohorts,” that is the HPTN071 sample. Unexpectedly, a significantly lower incidence was not observed in arm A relative to SOC. Importantly, rates of participation in the HPTN071 sample differed among population subgroups, for example men were underrepresented.

**Methods:**

To correct for underrepresented subgroups, PopART intervention effects are estimated in a population of interest, adults aged 18−44 in trial provinces, characterized with two nationally representative HIV‐focused surveys. The HPTN071 sample is weighted to match the population of interest by demographics and HIV risk factors. Risk of HIV acquisition is compared across arms, both in the trial population (unweighted) and the population of interest (weighted). Both (1) the risk of HIV acquisition between 1 and 3 years and (2) the risk of HIV acquisition by 3 years are compared.

**Results:**

In the trial population, estimated risk in arm A is, counterintuitively, slightly higher than SOC (Year 1−3 Risk Difference [RD]: 0.10%; 95% CI: −1.15%, 1.25%). After weighting, risk in arm A is lower than SOC in the population of interest (RD: −0.34%; 95% CI: −2.04%, 0.96%). Weighting also strengthened the estimated effect in arm B relative to SOC (unweighted RD: −0.66%, 95% CI: −1.88%, 0.46%; weighted RD: −1.18%, 95% CI: −2.85%, 0.15%). Weighted year 3 risk difference estimates indicated even stronger possible intervention effects: A versus SOC −0.83% (95% CI: −2.94%, 0.99%), B versus SOC −1.86% (95% CI: −3.80%, −0.09%).

**Conclusions:**

PopART interventions are estimated to be more protective in the population of interest than observed in the HPTN071 sample. These results partially explain the unexpected finding in arm A, providing further support for UTT strategies for HIV prevention. This analysis also highlights the importance of considering heterogeneous treatment effects among population subgroups when measuring the overall efficacy of HIV interventions.

## 1 INTRODUCTION

The HIV Prevention Trials Network (HPTN) 071 (PopART) study was a cluster‐randomized trial designed to evaluate universal testing and treatment (UTT) strategies for HIV prevention [1]. With UTT approaches, individuals with HIV are identified through testing and then treated to attain viral suppression and prevent HIV transmission [2, 3]. Conducted between 2013 and 2018, HPTN071 was the largest of four UTT trials conducted in sub‐Saharan Africa. These trials were instrumental in expanding UTT policies globally [4]. HPTN071 included three arms: (A) combination prevention with UTT; (B) combination prevention with universal HIV testing and antiretroviral therapy (ART) according to local guidelines; and (C) standard of care (SOC), with ART according to local guidelines and no combination prevention intervention or universal HIV testing.

PopART interventions were implemented in entire randomized communities, and their impact on HIV incidence was measured on sampled “Population Cohort” participants, that is the HPTN071 sample. Arm B had significantly lower HIV incidence from 1 to 3 years after baseline compared to SOC, but, surprisingly, arm A did not. The primary manuscript described the arm A findings as “unanticipated” and offered several possible explanations for the result, including potential differences in socio‐demographic characteristics and other factors across communities and possible selection bias [1]. Further work ruled out additional hypotheses related to sexual disinhibition [5], differential rates of migration [6], and lower ART retention and adherence [7].

A key strength of well‐conducted randomized trials is internal validity, which allows for valid comparisons of interventions within trial populations. External validity relates to how well trial results extend to other populations and may be a concern when trial samples differ from the populations where interventions will be applied [8, 9]. This can occur when recruitment procedures have varied success in enrolling individuals with different demographic characteristics and risk factors. For example, if recruitment and study visits occur during the day, it can be more challenging to enrol and retain individuals who are employed during the day, limiting their representation in the trial population. While HPTN071 used random sampling to recruit participants, non‐participation led to important differences in demographics and HIV risk factors between the HPTN071 sample, where intervention effects were measured, and the general population. Notably, HPTN071 sample members were more likely to be female and younger than the general population.

Generalizability methods can be used to improve external validity by extending the results of randomized trials to populations of interest [8]. Such methods typically require a representative sample of the population of interest. These methods are particularly beneficial in settings where the composition of the trial population and the population of interest differ by important characteristics for which the efficacy of the intervention also differs. For example, the composition of the HPTN071 sample and the general population differed by sex and age, and the PopART intervention was more effective in reducing HIV incidence among men than women and among older persons relative to younger persons [1]. In such settings, the overall efficacy of an intervention in the population of interest can differ in both magnitude and direction, sometimes substantially, from the effect observed in the trial sample.

The goal of this analysis was to determine whether the unexpected finding in HPTN071 could be explained by differences between the trial population and the population of interest. We used generalizability methods to estimate the effects of the PopART interventions in the population of interest, by weighting the HPTN071 sample using data from two nationally representative, HIV‐focused surveys conducted during the same timeframe as HPTN071. Because the individuals for whom the intervention was most effective (e.g. men and older individuals) were underrepresented in the HPTN071 sample, we hypothesized that estimates based on these generalizability methods would indicate greater efficacy of the PopART interventions relative to SOC than was reflected in the original trial analysis. This finding would affirm the benefits of UTT approaches and highlight the importance of estimating the efficacy of new HIV strategies in samples that well represent the populations where these strategies will ultimately be applied.

## 2 METHODS

### 2.1 Study design

We generalize HIV prevention results from HPTN071 to the population of interest, defined as adults aged 18−44 without HIV residing in urban areas within the five provinces where HPTN071 was conducted.

#### 2.1.1 HPTN071 (PopART)

HPTN071 included 21 urban communities in five provinces of Zambia and South Africa. Random samples of households were selected from each trial community for recruitment of the cohort in which HIV incidence was measured. Researchers visited selected households and randomly selected a single resident aged 18−44 for inclusion in the HPTN071 sample. Sample members were enrolled at baseline (*n* = 38,474), and additional sample members (*n* = 9827) were enrolled at 12 and 24 months due to lower than anticipated enrolment [1]. Household participation rates were slightly higher in arm B (73%) than in arms A and C (66% and 67%, respectively). Individual participation rates were comparable across arms (85% in arm A and 84% in arms B and C) [1]. Sample members completed surveys at baseline, 12, 24 and 36 months. Surveys included demographic, socio‐economic, HIV risk factor, diagnosis and treatment items. Blood samples were collected for HIV testing.

UTT interventions included HIV prevention and treatment components for all community residents. The combination HIV prevention intervention was delivered to individuals in randomized communities by community HIV care workers. This intervention included HIV counselling, rapid testing, support for linkage to care at government clinics and ART adherence for people with HIV, referrals for uncircumcised men without HIV for voluntary medical male circumcision, antenatal care for pregnant women with HIV and other health services. ART was provided at local government clinics to people with HIV in arm A communities regardless of their CD4 cell counts. For people in arm B and C communities, ART was provided for those with CD4 cell counts below 350 cells per microlitre initially, 500 cells per microlitre starting in 2014 and to all people with HIV starting in 2016. Thus, arms A and B had equivalent treatment policies from 2016 to 2018.

This analysis includes the 29,130 HPTN071 sample members enrolled at baseline who tested negative for HIV, 60% of the selected sample. Those enrolled later in the study were excluded because they did not have variables measured prior to the intervention. Up to 3 years of follow‐up are included. Additional details of the HPTN071 design and primary findings are reported elsewhere [1, 10].

#### 2.1.2 ZAMPHIA and SABSSMV

Two nationally representative HIV‐focused surveys were conducted during the same timeframe as HPTN071: the 2016 Zambia Population‐based HIV Impact Assessment survey (ZAMPHIA) [11] and the 2017 South African National HIV Prevalence, Incidence, Behaviour and Communication Survey V (SABSSMV) [12]. These surveys used multi‐stage probability sample designs to characterize the demographic, socio‐economic and HIV risk characteristics of individuals living in geographic areas where HPTN071 was conducted. In each survey, small geographic areas were formed, stratified by province and randomly selected. Within randomly selected areas, households were enumerated and randomly sampled. All age‐eligible individuals in sampled households were invited to participate. Those who participated could provide a blood sample for the biomarker survey, which included HIV testing. Participants also completed detailed questionnaires containing demographic, socio‐economic and HIV risk characteristics.

Participation rates were relatively high in each survey. In ZAMPHIA, 89% of eligible selected households participated, 86% of eligible individuals completed the interview and 90% of interviewed participants provided blood samples for biomarker testing [11]. In SABSSMV, 82% of eligible selected households participated, 94% of eligible individuals completed an interview and 61% provided blood samples for biomarker testing [12]. In both surveys, to minimize potential bias due to non‐participation, initial sampling weights were adjusted for non‐response for households, individuals, and for biomarker participation and were benchmarked to national census totals. Weight adjustments for non‐participation aim to make ZAMPHIA/SABSSMV nationally representative. Additional details regarding the ZAMPHIA and SABSSMV designs are provided elsewhere [11, 12].

ZAMPHIA/SABSSMV data were restricted based on age, province, urbanicity and HIV test status such that only those in the HPTN071 population of interest were included in the analytic sample. Sampling weights quantify the number of individuals in the population of interest represented by each survey participant. The 5076 ZAMPHIA/SABSSMV participants are representative of approximately 4.5 million adults in the population of interest.

### 2.2 Statistical analysis

#### 2.2.1 Variables of interest

HPTN071 evaluated PopART intervention efficacy on HIV incidence over a 3‐year period. For purposes of harmonization with the primary analysis in HPTN071, here the differences in HIV risk from 1 to 3 years after baseline for each intervention arm (A and B) relative to SOC (arm C) are estimated; this analysis excludes HIV acquisitions that occurred during the first year of follow‐up and thus we refer to this as the “modified intention to treat (ITT)” analysis. In addition, we estimated the differences in cumulative HIV risk at years 1 and 3; this analysis does not exclude HIV acquisitions that occurred during the first year of follow‐up and, therefore, is referred to as the “ITT” analysis. For both modified ITT and ITT analyses, the effect of the intervention arms combined relative to SOC was also estimated due to concordance in the interventions in the later years of the trial.

Consistent with the HPTN071 protocol, time to HIV acquisition was based on the estimated date of seroconversion. For non‐acute HIV acquisitions, the date of seroconversion was assigned as the midpoint of the last visit without HIV and the first visit with HIV. For acute infections, the seroconversion date was assigned to be the observed visit date when the acute infection was detected. Participants were censored at their last HIV‐negative visit if it occurred prior to 3 years, and were administratively censored at 3 years.

Covariate definitions were harmonized between HPTN071, ZAMPHIA and SABSSMV: sex and male circumcision (female, male‐circumcised, male‐uncircumcised), age group (18−24, 25−29, 30−34, 35−39, 40−44), educational attainment (none or primary, some secondary or more), alcohol use in the past 12 months (never, monthly or less, more than once per month), married or living together as married, currently employed, number of sexual partners in the last year (0, 1, 2+) and ever taken an HIV test (yes, no/don't know). Additional covariates used to adjust for loss to follow‐up in HPTN071 (described subsequently) were defined for only HPTN071 participants: dwelling unit type (single unit, multi‐unit, other), recreational drug use, nights spent outside the community in the past 3 months (0, 1−7, 8−30, 31−60, >60) and condom use at last sex within the last 12 months (yes, no).

#### 2.2.2 Analytic methods

Baseline characteristics of the HPTN071 sample were examined by arm and compared with the population of interest. To estimate the effect of the PopART interventions in the population of interest, generalizability methods were used to standardize the estimated intervention effect by covariates that (1) differed in distribution between the HPTN071 sample and the weighted ZAMPHIA/SABSSMV sample and (2) were expected to produce heterogeneous treatment effects. A detailed description of the generalizability methods applied in this analysis is presented in the Supplementary Material. Briefly, inverse odds of sampling weights were applied to upweight HPTN071 participants with covariate patterns more common in ZAMPHIA/SABSSMV and downweight HPTN071 participants with covariate patterns less common in ZAMPHIA/SABSSMV [13]. The weighted HPTN071 sample resembled the population of interest for the covariates (Supplementary Figure A1). To quantitatively assess the balance between the HPTN071 sample and the population of interest, standardized mean differences in covariates were computed before and after weighting. Next, inverse probability of censoring weights were estimated for HPTN071 participants to account for potentially informative dropout by trial arm and baseline covariates [14].

Overall weights for each HPTN071 participant were obtained by multiplying the inverse odds of sampling weights, the inverse probability of censoring weights and the inverse probability of the assigned treatment together. The estimated risk of HIV acquisition at each time point in each trial arm was estimated by the weighted empirical distribution function. Subsequently, the results from these analyses are referred to as “weighted” estimates. For comparison, an “unweighted” analysis was also conducted, where the risk of HIV acquisition was estimated using an unweighted Kaplan−Meier estimator. Risk differences are presented in the main text, and risk ratios are included in the Supplementary Material. Missing covariate data were handled using multiple imputation by chained equations with 50 imputed data sets. Confidence intervals were constructed using stratified bootstrap procedures for clustered data, which account for correlated data within trial communities.

Additional analyses aimed to identify the sources of differences between unweighted and weighted results, as outlined in the Supplementary Material.

Data management was conducted in SAS version 9.4 (Cary, NC), and statistical analyses were conducted in R version 4.3.1 (Vienna, Austria). Protocols were approved by ethics committees and other relevant institutional review boards for each study (HPTN071, ZAMPHIA and SABSSMV). Participants provided informed consent. This analysis is based on publicly available de‐identified data. The University of North Carolina at Chapel Hill internal review board (IRB) deemed this research not to constitute human subject research and did not require IRB approval.

## 3 RESULTS

Notable differences were present between HPTN071 participants and the population of interest represented by the ZAMPHIA/SABSSMV sample (Table 1). Females were overrepresented in HPTN071 relative to the population of interest (68−69% vs. 54%), as were younger persons (44−47% in HPTN071 aged 18−24 vs. 36% in the population of interest). Compared to the population of interest, HPTN071 participants reported lower use of alcohol more than once in the past month (9−15% vs. 25%) and had lower current employment (22−25% vs. 38%). Standardized mean differences (Supplementary Table A1) indicate relatively large discrepancies between HPTN071 participants and the population of interest prior to weighting with respect to sex and male circumcision, age category, alcohol use in the past month, current employment and the number of sexual partners in the past year. As expected, after weighting, the standardized mean differences were relatively small (0.10 or lower), indicating that weighting led to balance between the HPTN071 sample and the population of interest with respect to these covariates.

**Table 1:** Comparison of baseline characteristics of HPTN071 (PopART) participants (by arm) and population of interest.

	HPTN071 sample^a^			
Variable	A *n* = 9594	B *n* = 10,235	C (SOC) *n* = 9301	Population of interest (*n* = 5076)^b^
Sex and male circumcision (%)				
Female	69%	68%	68%	54%
Male, uncircumcised	16%	17%	18%	24%
Male, circumcised	15%	15%	14%	22%
Age category				
18−24	46%	44%	47%	36%
25−29	22%	21%	21%	20%
30−34	15%	15%	15%	19%
35−39	9%	11%	10%	14%
40−44	8%	9%	7%	11%
Educational attainment—Primary school or less	20%	17%	14%	15%
Alcohol use, past month				
Never	78%	69%	71%	59%
Monthly or less	13%	15%	20%	16%
More than once per month	9%	15%	9%	25%
Married	43%	39%	37%	38%
Currently employed	25%	29%	22%	38%
Number of sexual partners, past year				
0	28%	34%	28%	25%
1	68%	59%	65%	67%
2+	5%	7%	7%	8%
Ever had an HIV test	88%	82%	81%	80%

*Note*: Displayed percentages are among those without missing values. Across these eight covariates, missing data (by arm) ranged from 0.3% to 6.7% in the HPTN071 sample and 0.0−7.9% in the combined population of interest data.

Abbreviation: SOC, standard of care.

^a^ Includes population cohort members without HIV who enrolled at baseline.

^b^ Population of interest estimates are based on ZAMPHIA and SABSSMV studies; percentages are weighted by sampling weights.

Based on the unweighted analysis that did not account for covariate differences between the HPTN071 sample and the population of interest, the risk of HIV acquisition between years 1 and 3 (modified ITT) for participants in arm A was 3.08% versus 2.98% in the SOC arm (Risk Difference [RD]: 0.10%; 95% CI: −1.15%, 1.25%), indicating a *slightly higher* risk in arm A relative to SOC (Table 2). With weighting, the estimated risk between years 1 and 3 for participants in arm A was 2.50% versus 2.84% in the SOC arm (RD: −0.34%; 95% CI: −2.04%, 0.96%). That is, the estimated effect in the population of interest is in the originally hypothesized direction and further from the null than the unweighted estimate, though the confidence interval still includes the null. The modified ITT estimate for arm B relative to SOC is in the same direction for the unweighted and weighted analyses, but weighting strengthened the estimated effect (unweighted: RD: −0.66%, 95% CI: −1.88%, 0.46% vs. weighted: RD: −1.18%, 95% CI: −2.85%, 0.15%).

**Table 2:** Unweighted versus weighted cumulative risk of HIV acquisition and risk differences between arms.

	Cumulative risk of HIV acquisition (%)				Risk difference (%)		
	Arm A	Arm B	Arm A/B	Arm C (SOC)	Arm A versus SOC	Arm B versus SOC	Arm A/B versus SOC
Unweighted							
Yr 1	1.77	1.45	1.60	2.01	−0.24 (−1.21, 0.70)	−0.56 (−1.42, 0.31)	−0.41 (−1.30, 0.43)
Yr 3	4.85	3.77	4.27	4.98	−0.14 (−2.22, 1.83)	−1.21 (−3.14, 0.64)	−0.71 (−2.50, 1.12)
Yr 1 to Yr 3^*^	3.08	2.32	2.67	2.98	0.10 (−1.15, 1.25)	−0.66 (−1.88, 0.46)	−0.30 (−1.41, 0.73)
Weighted							
Yr 1	1.33	1.14	1.23	1.82	−0.49 (−1.35, 0.33)	−0.68 (−1.53, 0.17)	−0.59 (−1.35, 0.17)
Yr 3	3.83	2.80	3.30	4.66	−0.83 (−2.94, 0.99)	−1.86 (−3.80, −0.09)	−1.37 (−3.30, 0.41)
Yr 1 to Yr 3^*^	2.50	1.66	2.06	2.84	−0.34 (−2.04, 0.96)	−1.18 (−2.85, 0.15)	−0.78 (−2.39, 0.43)

*Note*: 95% confidence intervals for the risk difference are displayed in parentheses.

Abbreviation: SOC, standard of care.

* Change in risk from year 1 to year 3, that is Yr 3 − Yr 1.

Unweighted results for the modified ITT endpoint comparisons were similar to findings in the primary manuscript among the same subset of participants. When limited to participants enrolled at baseline, primary manuscript unadjusted IRRs were 1.04 (A vs. SOC) and 0.73 (B vs. SOC), which correspond to approximate year 1−3 risk differences of 0.09% (A vs. SOC) and −0.78% (B vs. SOC).

**Figure 1:**
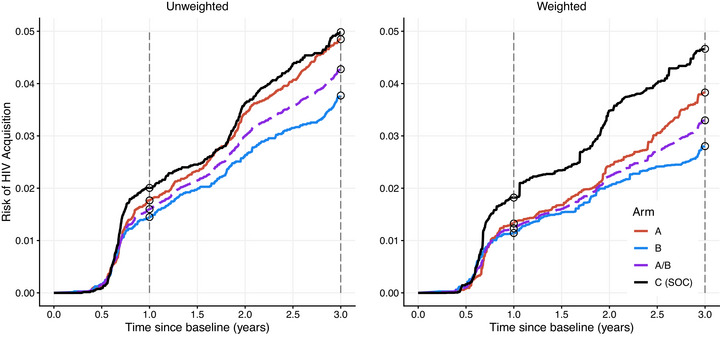
Estimated cumulative risk of HIV acquisition from baseline to year 3 by arm: unweighted versus weighted. *Note*: Estimates are based on 29,130 HPTN071 participants and 5076 ZAMPHIA/SABSSMV participants aged 18−44 in urban areas within five HPTN071 provinces. Weighted estimates account for differences between HPTN071 participants and the population of interest by sex and male circumcision, age, education, alcohol use, marital status, employment, number of sexual partners and HIV testing. Estimates are averaged across 50 multiply imputed datasets. Estimated cumulative risk in each arm at years 1 and 3 is circled. Abbreviation: SOC, standard of care.

When combining data from intervention arms A and B, the modified ITT effect estimates are in the same direction for the unweighted and weighted approaches, but weighting more than doubled estimated effects (unweighted: RD: −0.30%, 95% CI: −1.41%, 0.73% vs. weighted: RD: −0.78%, 95% CI: −2.39%, 0.43%). Notably, weighting reduced the estimated risk across all three arms at each time point (Figure 1).

**Figure 2:**
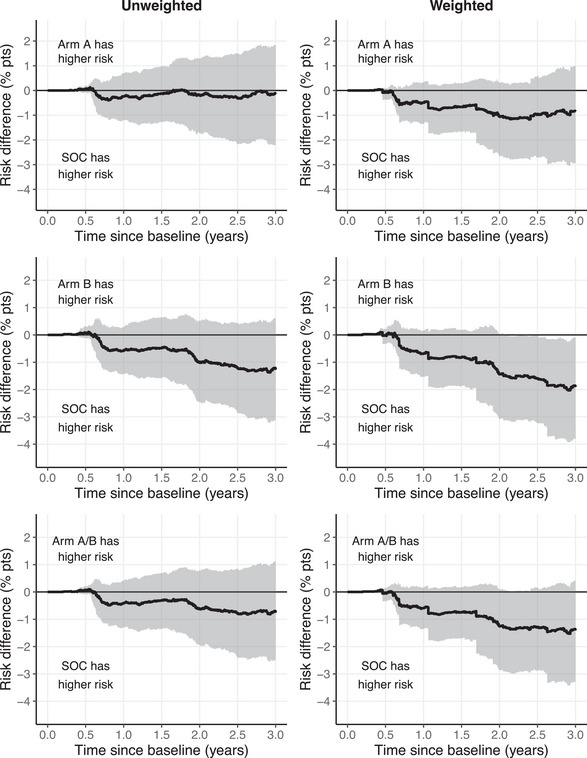
Estimated risk differences from baseline to year 3: unweighted versus weighted. *Note*: Estimates are averaged across 50 multiply imputed datasets. Pointwise 95% confidence intervals are shaded in grey. Abbreviation: SOC, standard of care.

Similar to the modified ITT results, estimated risk differences at years 1 and 3 that included HIV acquisitions in year 1 (i.e. ITT endpoint estimates) were stronger with weighting compared to unweighted (Table 1 and Figure 2). Notably, 3‐year risk differences were stronger for *both* unweighted and weighted analyses compared to the modified ITT approach. At 3 years, the weighted A to SOC risk difference (−0.83, 95% CI: −2.94%, 0.99%) is more than double the year 1−3 modified ITT risk difference estimates, and the weighted B to SOC risk difference is strengthened with a corresponding CI that excludes the null (−1.86%, 95% CI: −3.80%, −0.09%).

Risk ratios followed the same pattern as risk differences and are included in Supplementary Table A3.

Differences between HPTN071 participants and the population of interest with respect to sex and male circumcision were the driving force of differences between unweighted and weighted results for the arm A versus SOC comparison (Supplementary Table A2). In particular, by upweighting men (according to circumcision status) relative to women to reflect their composition in the population of interest and ignoring all other covariates, the intervention effect changed in both magnitude and direction (from 0.10% to −0.32%). This is due to the intervention's stronger efficacy among men compared to women. No single variable explained differences between unweighted and weighted results for the arm B versus SOC comparison (Supplementary Table A2).

## 4 DISCUSSION

HPTN071 was a key trial for evaluating the effectiveness of UTT strategies at a population level, but it had an unexpected non‐significant finding in arm A. HPTN071 may have faced challenges with external validity. Non‐participation led to notable differences between the HPTN071 sample and the population of interest where the interventions were applied. In particular, the HPTN071 sample underrepresented men relative to the population of interest. Here, generalizability methods were applied to account for differences between the HPTN071 sample and the population of interest using data from nationally representative surveys. The effects of both PopART interventions in the population of interest are estimated to be stronger than the primary HPTN071 findings [1].

The central strength of this analysis is the ability to leverage data from representative surveys to account for the underrepresentation of subgroups and estimate the effect of PopART interventions among all individuals in the areas where the trial was conducted. This work addresses a previously unexplained and unexpected finding in the largest UTT trial ever conducted, further strengthening the body of support for UTT approaches. While much focus in global HIV prevention has shifted to widespread Pre‐Exposure Prophylaxis (PrEP) rollout, this work is a reminder that reducing community viral load through UTT efforts reduces HIV transmission. Even in the age of PrEP, continued support for UTT programmes is essential for achieving global HIV reduction targets, as further supported by recent HIV risk assessment tools [15, 16].

HPTN071 was conducted between 2013 and 2018, which partially limits the direct impact of these findings on present‐day UTT policy decisions. Differences between the primary analysis and these findings emphasize the importance of considering issues surrounding external validity, particularly when heterogeneous treatment effects are suspected. When HIV prevention approaches differ in efficacy across population subgroups, comparing the composition of the trial population and the population where the intervention will be applied is critical. Overall estimates can change in both magnitude and direction when there are such heterogeneous treatment effects, making it appear that interventions are less (or more) beneficial than they will be in practice. Approaches for improving the representativeness of trial samples and planned secondary analyses that implement generalizability methods like those used here should be considered for wider adoption, when feasible.

In this analysis, ZAMPHIA and SABSSMV are assumed to represent the population of interest by using probability‐based sample designs and weight adjustments for non‐participation. These surveys could still suffer from non‐response bias if participants and non‐participants differ on characteristics not accounted for by weight adjustments. The additional weighting approaches applied in this analysis assume that all important covariates that (1) modify the efficacy of the interventions and differ in distribution between the population of interest and the HPTN071 sample and (2) inform participant dropout were accurately measured and included. Unfortunately, some risk factors for HIV acquisition and transmission, such as Herpes simplex virus type 2 [17], were not measured in ZAMPHIA/SABSSMV and thus could not be accounted for here. There may be other important variables that were not accounted for in this analysis, such as contextual factors that are difficult to measure but which influence trial participation and HIV risk. We assumed that there were no spillover effects across communities [18]. The unweighted methods used here differ from those used in the primary analysis, though estimates based on participants who enrolled at baseline were similar between the two approaches. Finally, results of HPTN071 were generalized to a broader population than HPTN071 communities, that is urban areas in trial provinces. Differences in unweighted and weighted results could be due to non‐participation in the HPTN071 sample or differences between HPTN071 communities and other urban areas within these provinces.

## 5 CONCLUSIONS

HPTN071 was one of four UTT trials conducted in sub‐Saharan Africa from 2012 to 2017. The other three trials were BCPP/Ya Tsie [19], SEARCH [20] and ANRS 12249/TasP [21]. While all four trials were designed to demonstrate population‐level benefits of UTT for reduced HIV incidence, their findings were mixed. Three trials had non‐significant findings in at least one intervention arm [4]. While prior explanations for these findings have emphasized changing ART eligibility criteria in control arms, migration in and out of trial communities and the relatively short periods of follow‐up [4], this work highlights the importance of accounting for differences between trial participants and the population of interest.

## COMPETING INTERESTS

JJE received funding from Gilead Sciences and ViiV Healthcare, paid to his institution, and consulting fees from Merck, ViiV Healthcare, Gilead Sciences and Abbvie. JJE also served on a DSMB or advisory board for TaiMed. All other authors declared no competing interests.

## AUTHOR CONTRIBUTIONS

BES‐S, PNZ, SRC and JKE conceptualized the overall study and were responsible for the acquisition of funding. DJD, HA, PB, RJH, SM and KZ conceptualized and oversaw data collection for the parent studies (HPTN071 and SABSSMV, respectively). BES‐S and PNZ analysed the data, oversaw the development of tables and figures, and accessed and verified the underlying data. All authors reviewed and edited the final manuscript and approved the final submission.

## FUNDING

This work was supported by the National Institutes of Health through grants K01AI182506, R01AI157758, K01AI177102, R21MH134750, R37AI029168, and P30AI50410 and by Cancer Research UK (PRCRPG‐Nov21∖100001).

## Supporting information



The supporting information contains a detailed description of the statistical methods and supplemental tables and figures for the analysis described in this manuscript.
Table A1. Standardized mean differences between HPTN071 (PopART) participants and population of interest pre‐ and post‐weighting
Table A2. Comparison of modified ITT risk differences between arms across methods
Table A3. Unweighted versus weighted cumulative risk of HIV infection and risk ratios between arms
Figure A1. Depiction of inverse odds of sampling weights

## Data Availability

This research is based on publicly available, de‐identified data obtained from the Harvard Dataverse (HPTN071), the Population‐Based HIV Impact Assessment Project (ZAMPHIA) and the Human Science Research Council (SABSSMV).
