# Supplementary material for: Examining the effect of universal testing and treatment strategies for HIV prevention in Zambia and South Africa: generalizing the results of the HPTN 071 (PopART) trial
Source: J Int AIDS Soc. 2025 Nov 26;28(12):e70062. doi: 10.1002/jia2.70062 (PMC12657112; doi:10.1002/jia2.70062)
Supplement: Supplementary file 1 — The supporting information contains a detailed description of the statistical methods and supplemental tables and figures for the analysis described in this manuscript. Table A1. Standardized mean differences between HPTN071 (PopART) participants and population of interest pre‐ and post‐weighting Table A2. Comparison of modified ITT risk differences between arms across methods Table A3. Unweighted versus weighted cumulative risk of HIV infection and risk ratios between arms Figure A1. Depiction of inverse odds of sampling weights [file JIA2-28-e70062-s001.docx]

**Examining the effect of universal testing and treatment strategies for HIV prevention
in Zambia and South Africa: generalizing the results of the HPTN 071 (PopART) trial**

**Supplementary material**

Bonnie E. Shook-Sa, Paul N. Zivich, Stephen R. Cole, Nora E. Rosenberg, Michael G. Hudgens, Deborah J. Donnell, Sizulu Moyo, Khangelani Zuma, Helen Ayles, Peter Bock, Joseph J. Eron, Richard J. Hayes,
Jessie K. Edwards

Contents

[Detailed Statistical Methods 2](#_Toc191917833)

[Multiple Imputation 2](#_Toc191917834)

[Estimating inverse odds of sampling weights 2](#_Toc191917835)

[Estimating inverse probability of censoring weights 3](#_Toc191917836)

[Estimating endpoints of interest 3](#_Toc191917837)

[Supporting Analyses 4](#_Toc191917838)

[Confidence interval construction 4](#_Toc191917839)

[Combining arms A and B 4](#_Toc191917840)

[Supplemental Tables 5](#_Toc191917841)

[**Table A1.** Standardized mean differences between HPTN071 (PopART) participants and population of interest pre- and post-weighting^1^ 5](#_Toc191917842)

[**Table A2.** Comparison of modified ITT^1^ risk differences between arms across methods 6](#_Toc191917843)

[**Table A3.** Unweighted versus weighted cumulative risk of HIV infection and risk ratios between arms 7](#_Toc191917844)

[Supplemental Figure 8](#_Toc191917845)

[**Figure A1.** Depiction of inverse odds of sampling weights 8](#_Toc191917846)

## Detailed Statistical Methods

This appendix provides a more detailed description of the statistical methods. Throughout the appendix, the combined ZAMPHIA/SABSSMV data are referred to as the “population-based survey sample.”

### Multiple Imputation

Missing covariates were imputed using multiple imputation by chained equations[1] with 50 imputation datasets following 5 burn-in iterations using the *mice* function in R[2]. Separate imputation models were fit for the HPTN071 sample and the population-based survey sample. The HPTN071 imputation model included community, event indicator, time to event, the harmonized covariates, and the additional covariates used to adjust for loss to follow-up in HPTN071 (defined in the main text). Treatment assignment was not included in the imputation model due to collinearity with the community variable. The population-based survey sample imputation model included sampling weight, province (Copperbelt, Central, Lusaka, and Southern provinces in Zambia or Western Cape province in South Africa), and the harmonized covariates. Sample design features (sampling weight and province) were included in the imputation model for the population-based survey sample based on established guidance regarding multiple imputation for complex survey data[3, 4].

### Estimating inverse odds of sampling weights

Inverse odds of sampling weights were estimated to generalize HPTN071 findings to the population of interest represented by the population-based survey sample. To mimic the HPTN071 design, separate sampling models were fit for each of the 21 HPTN071 communities. The details of these models are subsequently described.

Within each multiply imputed dataset and HPTN071 community, data from the HPTN071 sample and the population-based survey sample were stacked and inverse odds of sampling weights[5] were estimated from sample weighted logistic regression models that predicted the probability of being in the population of interest conditional on the harmonized covariates. For population-based survey sample members, the sampling weights were divided by 21 such that resulting inverse odds of sampling weights were expected to sum to the population of interest size when data were subsequently pooled across the 21 communities. Consistent with previously developed generalizability methods for survey data[6], when estimating parameters of the sampling models, population-based survey sample members were weighted by the adjusted sampling weights while HPTN071 participants received a weight of one.

Within each multiply imputed dataset and HPTN071 community, the predicted probability of being in the population of interest, $\pi_{Si}$, was estimated from the corresponding fitted model for each participant $i$. The inverse odds of sampling weight was defined as ${IOSW}_{i}=\pi_{Si}/(1-\pi_{Si})$ for each HPTN071 participant. After model fitting, data were limited to HPTN071 participants and pooled across all 21 HPTN071 communities. The overall sum of inverse odds of sampling weights was compared with the estimated size of the population of interest, i.e., the sum of the population-based survey sampling weights. The ratio of inverse odds weight sums to population of interest sizes was expected to be approximately one and ranged from 1.02-1.03 across the 50 imputed datasets.

To assess how well inverse odds of sampling weights provided balance between weighted HPTN071 participants and the population of interest for the eight covariates of interest, standardized mean differences[7] in sampling model covariates were computed before and after weighting by the inverse odds of sampling weights. The *smd* function in R[8] was used to estimate standardized mean differences. Pre-weighted differences were based on weights of one for HPTN071 participants and sampling weights for population-based survey sample members. Post-weighted differences were based on inverse odds of sampling weights for HPTN071 participants and sampling weights for population-based survey sample members. Standardized mean differences were averaged across the 50 imputed datasets.

### Estimating inverse probability of censoring weights

Within each imputed dataset, time-varying inverse probability of censoring weights were estimated for HPTN071 participants to account for dropout that is potentially informed by trial arm and covariates. The probability of remaining uncensored ($\pi_{Ci}$) at observed event times $T_{i}^{*}$was estimated from a Cox proportional hazard model along with the Breslow estimator for the cumulative hazard[9, 10]. This model was stratified by trial arm and included the harmonized covariates from the sampling model, as well as the additional baseline covariates defined for HPTN071 participants, as described in the main text. The inverse probability of censoring weights were then defined at $T_{i}^{*}$ as ${IPCW}_{i}={1/\pi}_{Ci}$.

### Estimating endpoints of interest

Inverse probability of treatment weights were defined for each HPTN071 participant based on known randomization probability of each treatment (1/3), i.e. ${IPTW}_{i}=\left( \frac{1}{3} \right)^{-1}= 3$. Overall weights for each HPTN071 participant were obtained by multiplying the inverse odds of sampling weights, the inverse probability of censoring weights, and the inverse probability of treatment weights such that ${WT}_{i}={IOSW}_{i}\times{IPCW}_{i}\times{IPTW}_{i}$. The estimated risk of HIV acquisition at each timepoint in each trial arm was estimated by the weighted empirical distribution function. That is, within each multiply imputed dataset, risk at time $t$ in arm $a$ was estimated by:

$$\hat{R}^{a}\left( t \right)=\frac{1}{\hat{n}_{TP}}\left[ \sum_{i=1}^{n} I\left( A_{i}=a \right)I\left( T_{i}^{*}\leq t \right)\delta_{i}\times{WT}_{i} \right] \mathrm{for} a\in\left\{ 1,2,3 \right\},$$

where $n=29,130$ (the number of HPTN071 participants), $A_{i}$ represents the assigned treatment arm, $T_{i}^{*}$ is the observed time to event, $\delta_{i}=1$ indicates seroconversion (versus $\delta_{i}=0$ indicating censoring), and ${WT}_{i}$ represents the overall HPTN071 participant weight described above. The estimated size of the population of interest is the denominator of the estimated risk and is calculated as the sum of the inverse odds of sampling weights for all HPTN071 participants, i.e., $\hat{n}_{TP}=\sum_{i=1}^{n} {IOSW}_{i}$.

Risk differences were estimated from the weighted risk functions for each arm. For the modified ITT endpoint (the difference in risk from years 1 to 3 between arms), the estimator is defined by $\hat{\Delta}_{I-C}=\left\{ \hat{R}^{I}\left( 3 \right)-\hat{R}^{I}\left( 1 \right) \right\}-\left\{ \hat{R}^{SOC}\left( 3 \right)-\hat{R}^{SOC}\left( 1 \right) \right\}$, where $I$ indicates the intervention arm ($I \epsilon\left\{ A, B \right\}$) and time in parentheses is indexed in years. In ITT analyses, risk differences of HIV acquisition at years 1 and 3 are estimated by $\hat{RD}_{I-C,t}=\hat{R}^{I}\left( t \right)-\hat{R}^{SOC}\left( t \right)$ for at $t \epsilon\left\{ 1,3 \right\}$. Risk curves and twister plots[11] were constructed by plotting the estimated risks and risk differences, respectively, at each timepoint between baseline and year 3.

For comparison, an unweighted analysis was conducted, where the risk of HIV acquisition at each timepoint in each arm was estimated using an unweighted Kaplan-Meier estimator that assumed noninformative dropout conditional on trial arm. Both weighted and unweighted results were averaged across the 50 imputed datasets.

### Supporting Analyses

Additional analyses aimed to identify the sources of differences between unweighted and weighted results. To distinguish the effects of censoring weights (which account for loss to follow-up within the HPTN071 sample) from inverse odds of sampling weights (which account for differences between the HPTN071 sample and the population of interest), the modified ITT analysis was repeated without inverse odds of sampling weights. To determine which covariates in the sampling model were most influential, the modified ITT analysis was repeated eight additional times, each with only a single covariate included in the sampling models.

### Confidence interval construction

For each estimate of interest, corresponding 95% confidence intervals were constructed from a stratified bootstrap procedure for clustered data with 2000 bootstrap replications.

For each bootstrap replicate, HPTN071 sample data were stratified by arm and seven communities were independently resampled with replacement from each of the three arms. Consistent with recommendations for bootstrap sampling with clustered data, all participants were retained within selected clusters[12]. The Rao-Wu rescaling bootstrap, designed for complex survey data, was applied to the population-based survey sample[13, 14]. After selection of separate bootstrap samples for the HPTN071 sample and the population-based survey sample, they were stacked for subsequent analyses.

Consistent with prior recommendations regarding combining bootstrap and multiple imputation methods with congenial models[15], multiple imputation was conducted independently within each of the 2000 bootstrap samples and the previously described analyses were subsequently replicated. Note HPTN071 communities sampled more than once were treated as independent clusters in subsequent analyses.

Percentile-based confidence intervals were derived from the distribution of bootstrap sample estimates. That is, the 2.5% and 97.5% percentiles of each estimate across the 2000 bootstrap replicates were the lower and upper 95% confidence interval limits, respectively.

### Combining arms A and B

Because the HPTN071 intervention arms were identical starting in 2016, results were also presented with arms A and B combined. All previously described methods were repeated, but with arms A and B treated as the same intervention. That is, censoring models were fit with the collapsed intervention as a stratification factor and $\hat{R}^{a}\left( t \right)$ was estimated for $a\in\left\{ 1\vee2,3 \right\}$. Note the overall weights ${WT}_{i}$ for HPTN071 participants in arms A and B assumed a treatment weight of ${IPTW}_{i}=\left( \frac{2}{3} \right)^{-1}$, which represents the inverse probability of treatment assignment to either arm A or B. For confidence interval construction, arms A and B were again treated as representing the same intervention.

## Supplemental Tables

### **Table A1.** Standardized mean differences between HPTN071 (PopART) participants and population of interest pre- and post-weighting^1^

|  | **Pre-weighting** | **Post-weighting** |
| --- | --- | --- |
| Sex and male circumcision | 0.35 | 0.10 |
| Age category | 0.24 | 0.08 |
| Educational attainment | 0.07 | 0.05 |
| Alcohol use, past month | 0.38 | 0.03 |
| Married | 0.02 | 0.08 |
| Currently employed | 0.30 | 0.01 |
| Number of sexual partners, past year | 0.15 | 0.10 |
| Ever had an HIV test | 0.07 | 0.08 |

**^1^** Standardized mean differences (SMDs) between the weighted population-based survey sample and the HPTN071 sample (unweighted [pre] and with inverse odds of sampling weights [post]). Presented SMDs are averaged across 50 imputed datasets. Note differences greater than 0.1 are considered to be indicative of imbalance[7].

### **Table A2.** Comparison of modified ITT^1^ risk differences between arms across methods

|  | **A vs SOC** | **B vs SOC** |
| --- | --- | --- |
| Unweighted analysis (Table 2 results) | 0.10% | -0.66% |
| Censoring weights and sampling weights, full model (Table 2 results) | -0.34% | -1.18% |
| Censoring weights, no sampling weights | 0.14% | -0.41% |
| Censoring weights and sampling weights, univariate sampling models |  |  |
| Sex and male circumcision | -0.32% | -0.83% |
| Age category | -0.14% | -0.87% |
| Educational attainment | -0.12% | -0.81% |
| Alcohol use, past month | -0.18% | -0.96% |
| Married | 0.17% | -0.71% |
| Currently employed | 0.04% | -0.68% |
| Number of sexual partners, past year | 0.09% | -0.76% |
| Ever had an HIV test | -0.05% | -0.90% |

**^1^** Risk differences estimate differences between arms in the change in risk from year 1 to year 3.
Note: SOC=standard of care. For each estimated risk difference, SOC is referent.

### **Table A3.** Unweighted versus weighted cumulative risk of HIV acquisition and risk ratios between arms

|  | Cumulative Risk of HIV Acquisition (%) | | | | Risk Ratio | | |
| --- | --- | --- | --- | --- | --- | --- | --- |
|  | Arm A | Arm B | Arm A/B | Arm C (SOC) | Arm A vs SOC | Arm B vs SOC | Arm A/B vs SOC |
| Unweighted |  |  |  |  |  |  |  |
| Yr 1 | 1.77 | 1.45 | 1.60 | 2.01 | 0.88 (0.49, 1.48) | 0.72 (0.44, 1.21) | 0.80 (0.51, 1.30) |
| Yr 3 | 4.85 | 3.77 | 4.27 | 4.98 | 0.97 (0.61, 1.49) | 0.76 (0.47, 1.17) | 0.86 (0.59, 1.29) |
| Yr 1 to Yr 3^*^ | 3.08 | 2.32 | 2.67 | 2.98 | 1.03 (0.67, 1.54) | 0.78 (0.47, 1.21) | 0.90 (0.61, 1.32) |
| Weighted |  |  |  |  |  |  |  |
| Yr 1 | 1.33 | 1.14 | 1.23 | 1.82 | 0.73 (0.43, 1.25) | 0.63 (0.35, 1.14) | 0.68 (0.44, 1.14) |
| Yr 3 | 3.83 | 2.80 | 3.30 | 4.66 | 0.82 (0.50, 1.29) | 0.60 (0.36, 0.97) | 0.71 (0.46, 1.12) |
| Yr 1 to Yr 3^*^ | 2.50 | 1.66 | 2.06 | 2.84 | 0.88 (0.50, 1.50) | 0.58 (0.31, 1.08) | 0.73 (0.43, 1.24) |

**^*^**Change in risk from year 1 to year 3, i.e., Yr 3 $-$ Yr 1
Note: SOC=standard of care. 95% confidence intervals for the risk ratio are displayed in parentheses.

## Supplemental Figure

### **Figure A1.** Depiction of inverse odds of sampling weights


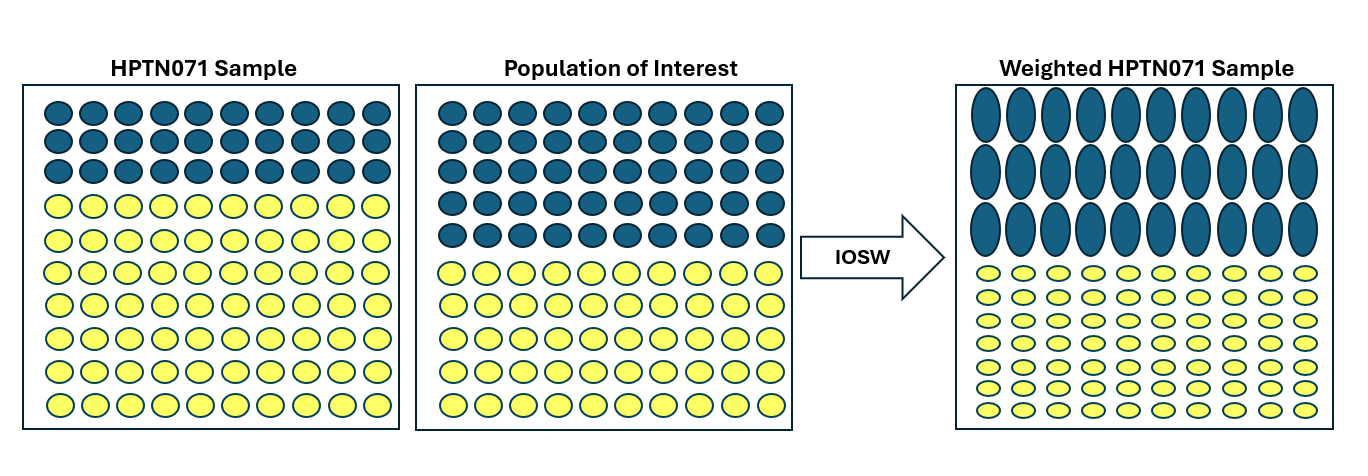


Note: Yellow dots represent women and blue dots represent men. In this example, men represent only 30% of the HPTN071 sample but 50% of the population of interest. Inverse odds of sampling weights (IOSW) re-weight the HPTN071 sample (up-weighting men and down-weighting women) such that women and men both account for 50% of the weighted population.

**References**

1. White IR, Royston P, Wood AM. Multiple imputation using chained equations: issues and guidance for practice. Statistics in Medicine. 2011;30(4):377-99.

2. van Buuren S, Groothuis-Oudshoorn K, Robitzsch A, Vink G, Doove L, Jolani S. Package ‘mice’. Computer software. 2015;20.

3. Rubin DB. Multiple imputation after 18+ years. Journal of the American Statistical Association. 1996;91(434):473-89.

4. Berglund P, Heeringa SG. Multiple imputation of missing data using SAS: SAS Institute; 2014.

5. Westreich D, Edwards JK, Lesko CR, Stuart E, Cole SR. Transportability of trial results using inverse odds of sampling weights. American Journal of Epidemiology. 2017;186(8):1010-4.

6. Ackerman B, Lesko CR, Siddique J, Susukida R, Stuart EA. Generalizing randomized trial findings to a target population using complex survey population data. Statistics in Medicine. 2021;40(5):1101-20.

7. Zhang Z, Kim HJ, Lonjon G, Zhu Y. Balance diagnostics after propensity score matching. Annals of Translational Medicine. 2019;7(1).

8. Saul B. smd: Compute Standardized Mean Differences. 2024.

9. Cox DR. Regression models and life-tables. Journal of the Royal Statistical Society. Series B (Methodological). 1972;34(2):187-220.

10. Breslow N. Discussion of the paper by DR Cox cited below. Journal of the Royal Statistical Society, Series B. 1972;34:187-220.

11. Zivich PN, Cole SR, Breskin A. Twister plots for time-to-event studies. American Journal of Epidemiology. 2021;190(12):2730-1.

12. Field CA, Welsh AH. Bootstrapping clustered data. Journal of the Royal Statistical Society Series B: Statistical Methodology. 2007;69(3):369-90.

13. Rao JN, Wu C. Resampling inference with complex survey data. Journal of the American Statistical Association. 1988;83(401):231-41.

14. Rao J, Wu C, Yue K. Some recent work on resampling methods for complex surveys. Survey Methodology. 1992;18(2):209-17.

15. Li CX, Zivich PN. Invited Commentary: Mixing multiple imputation and bootstrapping for variance estimation. American Journal of Epidemiology. 2024:kwae065.
